# Supplementary material for: Alterations in Circulating T-Cell Subsets with Gut-Homing/Residency Phenotypes Associated with HIV-1 Status and Subclinical Atherosclerosis
Source: Cells. 2025 Nov 4;14(21):1732. doi: 10.3390/cells14211732 (PMC12607611; doi:10.3390/cells14211732)
Supplement: Supplementary file 1 [file cells-14-01732-s001.zip › Suppl Files EMG 20251103/EMG JD Suppl. Figs 1-3 20251103.pdf]

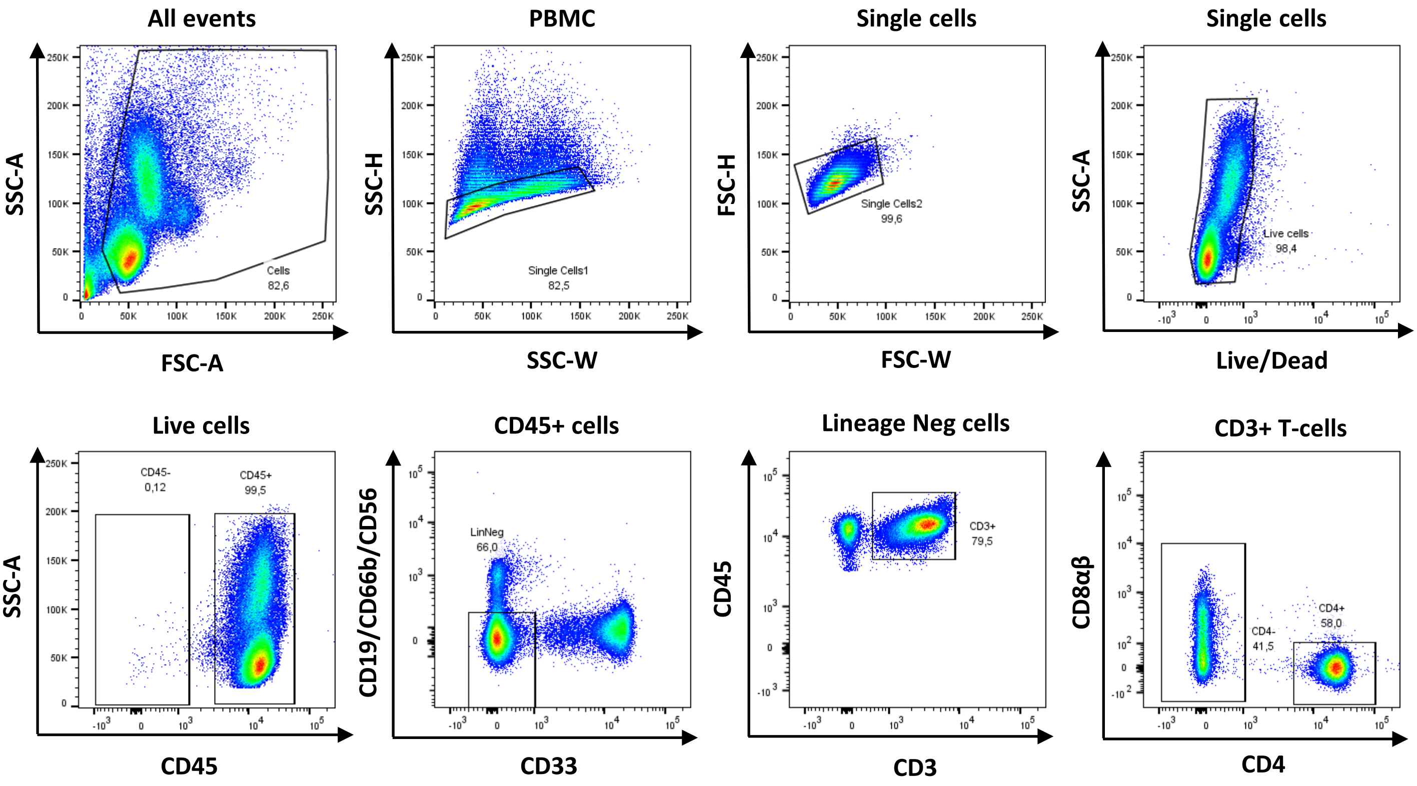

Supplemental Figure 1 (related to Figures 1-6): General gating strategy for flow cytometry analysis of PBMCs of study participants. PBMCs from HIV$^+$ART (n=22) and HIV$^-$ (n=20) participants with known CVD status, measured as the total plaque volume (TPV; mm^3) of the coronary artery atherosclerosis, were stained with the Fixable Viability Stain 575V for dead cell exclusion and with a cocktail of abs that allowed the exclusion of other lineage cells (CD33, CD56, CD19 and CD66b), and the identification of CD45$^+$ hematopoietic cells. Among CD45$^+$ cells, CD3$^+$ T-cells were further differentiated into CD4$^+$ and CD8αβ$^+$ T-cells.

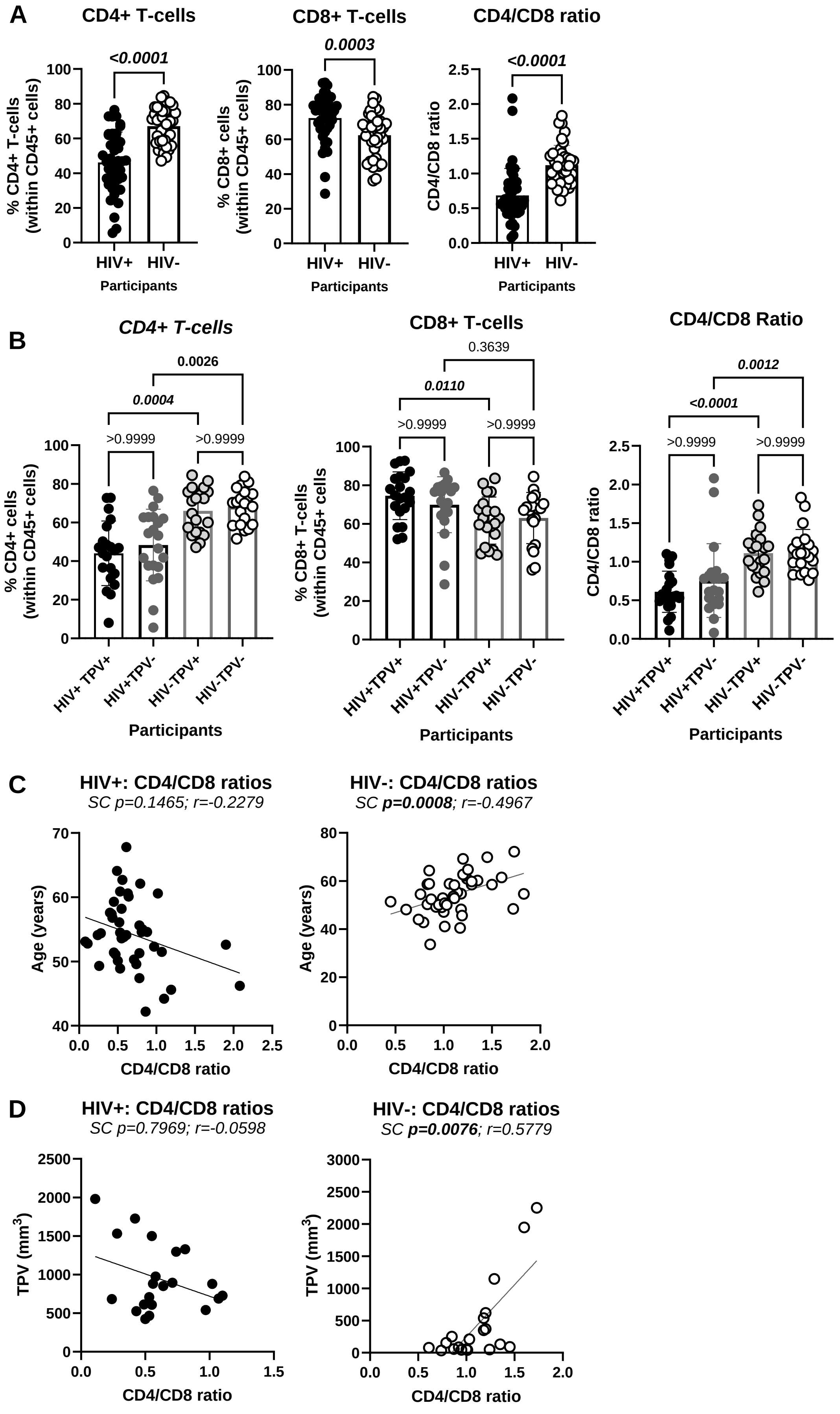

Moreira Gabriel/Dias et al., Supplemental Figure 2
Relative to Figure 1

Supplemental Figure 2 (related to Figure 1): Frequency of CD4+ and CD8+ T-cells in HIV+ *versus* HIV- participants relative to age and subclinical CVD. PBMCs from study participants were identified as per Supplemental Figure 1. Shown are **(A)** the frequency of $CD4^+$ T-cells, $CD8^+$ T-cells and the CD4/CD8 ratio relative to the HIV status; **(B)** the characterization of $CD4^+$ T-cells, $CD8^+$ T-cells and CD4/CD8 ratio relative to the presence of subclinical CVD; **(C)** the correlation between age and the CD4/CD8 ratio in HIV^+ **(left panel)** and HIV^- **(right panel)** participants; and **(D)** the correlation between TPV and the CD4/CD8 ratio in HIV^+ **(left panel)** and HIV^- **(right panel)** participants. Mann-Whitney p-values, as well as Spearman correlation p and r values, are indicated on the graphs.

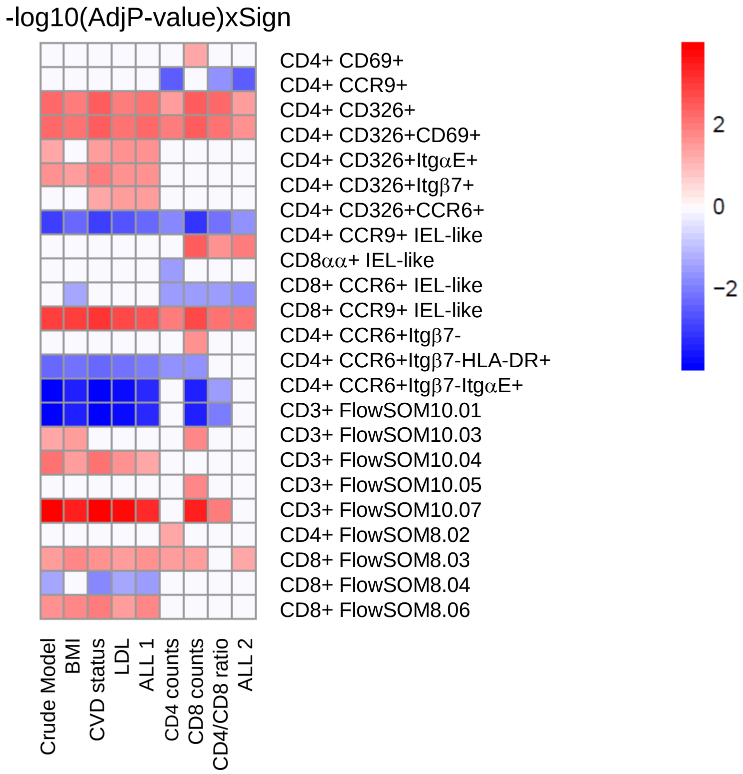

Supplemental Figure 3 (related to Supplemental Table 1): Multivariate regression model identified novel immunological predictors of HIV-1 status. Heatmap illustrate the subsets of T-cells that were identified in Supplemental Table 1 as statistically significant predictors of the HIV-1 status in the crude model, or upon adjusting for parameters identified as statistically different between HIV+ART and HIV- groups (Table 1), as follows: BMI, CVD status, LDL, ALL 1 (BMI, CVD status, LDL), as well as CD4 counts, CD8 counts, CD4/CD8 ratios, and ALL 2 (CD4 counts, CD8 counts, CD4/CD8 ratios). Adjusted p-values (-log10) are indicated as a red and blue gradient for positively (Sign=1) and negatively associated predictors (Sign=-1), respectively.
